# Altered mechanotransduction in adolescent idiopathic scoliosis osteoblasts: an exploratory in vitro study

**DOI:** 10.1038/s41598-022-05918-0

**Published:** 2022-02-03

**Authors:** Niaz Oliazadeh, Kristen F. Gorman, Mohamed Elbakry, Alain Moreau

**Affiliations:** 1 Viscogliosi Laboratory in Molecular Genetics of Musculoskeletal Diseases, Saint-Justine University Hospital Research Center, room 2.17.027, 3175 Cote-Ste-Catherine Road, Montreal, QC H3T 1C5 Canada; 2 Department of Biochemistry and Molecular Medicine, Faculty of Medicine, Université de Montréal, Montreal, QC Canada; 3 Department of Biological Sciences, California State University, Chico, CA 95929 USA; 4 Biochemistry Division, Chemistry Department, Faculty of Science, Tanta University, Tanta, Egypt; 5 Department of Stomatology, Faculty of Dentistry, Université de Montréal, Montreal, QC Canada

**Keywords:** Biochemistry, Cell biology

## Abstract

Adolescent idiopathic scoliosis (AIS) is the most prevalent pediatric spinal deformity. We previously demonstrated elongated cilia and an altered molecular mechanosensory response in AIS osteoblasts. The purpose of this exploratory study was to characterize the mechanosensory defect occurring in AIS osteoblasts. We found that cilia length dynamics in response to flow significantly differ in AIS osteoblasts compared to control cells. In addition, strain-induced rearrangement of actin filaments was compromised resulting in a failure of AIS osteoblasts to position or elongate in function of the bidirectional-applied flow. Contrary to control osteoblasts, fluid flow had an inhibitory effect on AIS cell migration. Moreover, flow induced an increase in secreted VEGF-A and PGE2 in control but not AIS cells. Collectively our data demonstrated that in addition to the observed primary cilium defects, there are cytoskeletal abnormalities correlated to impaired mechanotransduction in AIS. Thus, we propose that the AIS etiology could be a result of generalized defects in cellular mechanotransduction given that an adolescent growing spine is under constant stimulation for growth and bone remodeling in response to applied mechanical forces. Recognition of an altered mechanotransduction as part of the AIS pathomechanism must be considered in the conception and development of more effective bracing treatments.

## Introduction

Adolescent Idiopathic Scoliosis (AIS) is a complex pediatric disease involving abnormal three-dimensional spinal curvatures of unknown cause. At the clinical level, a wide range of curve patterns and magnitudes illustrates AIS’s phenotypic heterogeneity. In the most severe cases, scoliosis is accompanied with rib cage deformity that can cause serious health issues such as pulmonary and cardiac distress. Clinically, idiopathic scoliosis is broadly categorized by the age when a curve onset is first noted. Adolescent idiopathic scoliosis is the most prevalent type of idiopathic scoliosis affecting an average of 2–4% of children aged 10 to 16 years old with a potential of progression during the rapid phase of growth^1,2^.

Despite decades of research into the etiology of AIS, the pathomechanism underlying this condition is still poorly understood^3^. Although a genetic basis is acknowledged, genetic heterogeneity coupled with the clinical variability of AIS have hindered our understanding of its biological basis^4,5^. Genetic predispositions, hormonal imbalance, neurological disorders and environmental factors have all been suggested to play a role in disease onset and scoliosis progression as well as in the elaboration of specific spinal curve patterns (reviewed in^6^).

AIS occurs during the pubertal growth spurt, and curve progression usually stabilizes at skeletal maturity^7^. Because the deformity presents clinically in the tissues of bone, cartilage, and muscle, idiopathic scoliosis is considered primarily as a musculoskeletal disease, although other physiological systems are implicated^6^. Considering the fact that tissues of the musculoskeletal system are load bearing, biomechanics is an important factor in the pathogenesis of the disease. Traditionally, the discipline of biomechanics has been applied to AIS at the anatomical level. For example, how a growing scoliotic spine can lose its mechanical stability, resulting in deformation of vertebral bodies, which could potentially induce compensatory curves and thus deformity progression^8^. Regardless of underlying etiological factors, the importance of biomechanics in the pathophysiology of AIS is well established, as is reflected in non-surgical treatment approaches (e.g. bracing, physical therapies)^9^. For AIS, understanding the physiological/molecular responses to mechanical stimuli can provide novel insights regarding its etiology, and holds many possibilities for improving the current therapeutic approaches and developing novel personalized options.

Our previous work showed that without mechanical stimulation, the primary cilia in AIS patient-derived osteoblasts is longer than the primary cilia in osteoblasts derived from non-scoliotic bone. Furthermore, we used a gene expression assay to demonstrate that these AIS cells are less responsive to mechanical stimulation^10^. Our comparative analysis of exomes indicated an enrichment for rare variants in genes involved in mechanotransduction and/or ciliogenesis among AIS patients^10^. Evidence that idiopathic scoliosis is associated with cellular biomechanical defects is further supported by several AIS genetic studies. These studies either corroborate a ciliopathy^11,12^, or implicate other mechanosensitive features such as subtle impairments of the extracellular matrix^13^. Taken together, recent evidence supports a disturbed cellular mechanosensory system in AIS.

The primary cilium is a dynamic mechanosensory organelle that can change length under varying circumstances allowing the cell to adjust its behavior in proportion to the environmental changes^14^. Molecular pathways involved in mechanosensation are responsible for cellular awareness of spatial orientation, occupation, and motility^15,16^. In this exploratory study, we investigated whether osteoblasts from AIS patients display different ciliary dynamics in response to oscillatory fluid flow. Then, we evaluated cellular behaviors such as migration, elongation and orientation in response to flow. In our previous study, we showed that the most dramatic difference in ciliary length between AIS patients and control-derived osteoblasts was before cellular starvation and at 24 h post-starvation. Considering that actin polymerization inhibitors induce longer cilia and facilitate ciliogenesis independently of starvation^17^, in this study we also investigated flow-induced actin rearrangement as well as changes in vascular endothelial growth factor (VEGF) and prostaglandin E2 (PGE2) secretion.

## Results

### Cilia length is differentially adjusted in AIS osteoblasts in response to fluid flow

**Figure 1:**
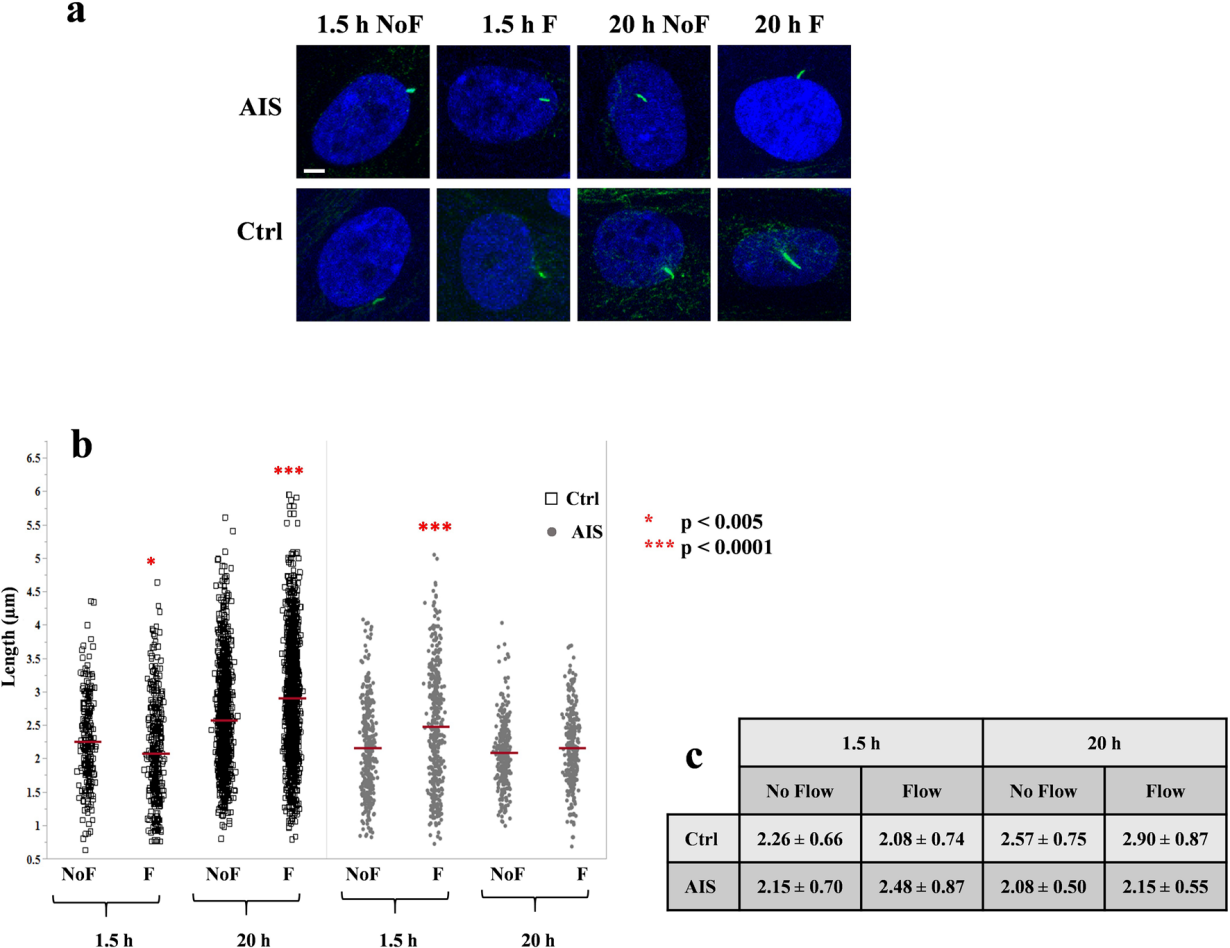
Flow induced primary cilia length adjustment is impaired in AIS osteoblasts. (**a**) Immunofluorescence micrographs of cilia using acetylated α-Tubulin (green) and Hoescht (blue), in AIS and control osteoblasts under static or mechanically stimulated conditions. White scale bar, 5 µm. (**b**) The dynamics of cilia length adjustment in response to flow application (F) in comparison with their static counterparts (NoF) in IS and controls. n = 4 per group, 25 fields of view, red bars represent the mean. Statistical significance was determined by Student T-test using JMP-14. (**c**) Values of the mean and standard deviation are summarized in this table.

Normal osteoblasts modify the length of their cilia in response to the intensity and duration of applied mechanical stimulation. This adaptive response is part of a biological regulatory process that allows the cells to adjust their mechanosensory structures proportionally to accommodate the mechanical challenge^18^. We investigated if the elongated cilia previously observed in stationary AIS osteoblasts behaves differently (compared to similar cells obtained from healthy subjects) after applying 1 Pa fluid flow shear stress for a short (1.5 h) or long (20 h) duration. We found that control cells reduced the length of their cilia significantly after 1.5 h of flow application (mean decrease of 8%, *P* < 0.005) while under long term flow application (20 h) their cilia length increased significantly (mean increase of 13.2%, *P* < 0.0001). Conversely, in AIS osteoblasts, short-term flow application induced an increase in the length of their cilia (mean increase of 13.3%, *P* < 0.0001), while long-term flow application had no significant effect (Fig. 1). Of note, the length of cilia in absence of flow application for 1.5 h and 20 h (NoF 1.5 h and NoF 20 h) in AIS samples was shorter (2.15 ± 0.70 µm and 2.08 ± 0.50) when compared to control osteoblasts (2.26 ± 0.66 µm and 2.57 ± 0.75 µm) but only the 20 h comparison reached a statistical significance (*P* < 0.0001) (Fig. 1c). Previously, we reported an increased length in cilia under no flow condition in AIS osteoblasts that was consistent up to 72 h of ciliary growth, in ciliogenesis media. In the current experiments, the cilia were measured post-ciliogenesis, after being transferred to regular media for 1.5 h or 20 h. In both cases, the observations further support an impaired regulation of cilia length in AIS that could contribute to the reported abnormal mechanosensory behaviours. Consequently, in order to minimize the effects of cilia length discrepancy at the starting point of our functional experiments, we chose the 48 h starvation point for ciliogenesis that previously showed lesser length variations between the two groups, as previously reported^10^.

### Flow induced actin remodeling is impaired in AIS osteoblasts

**Figure 2:**
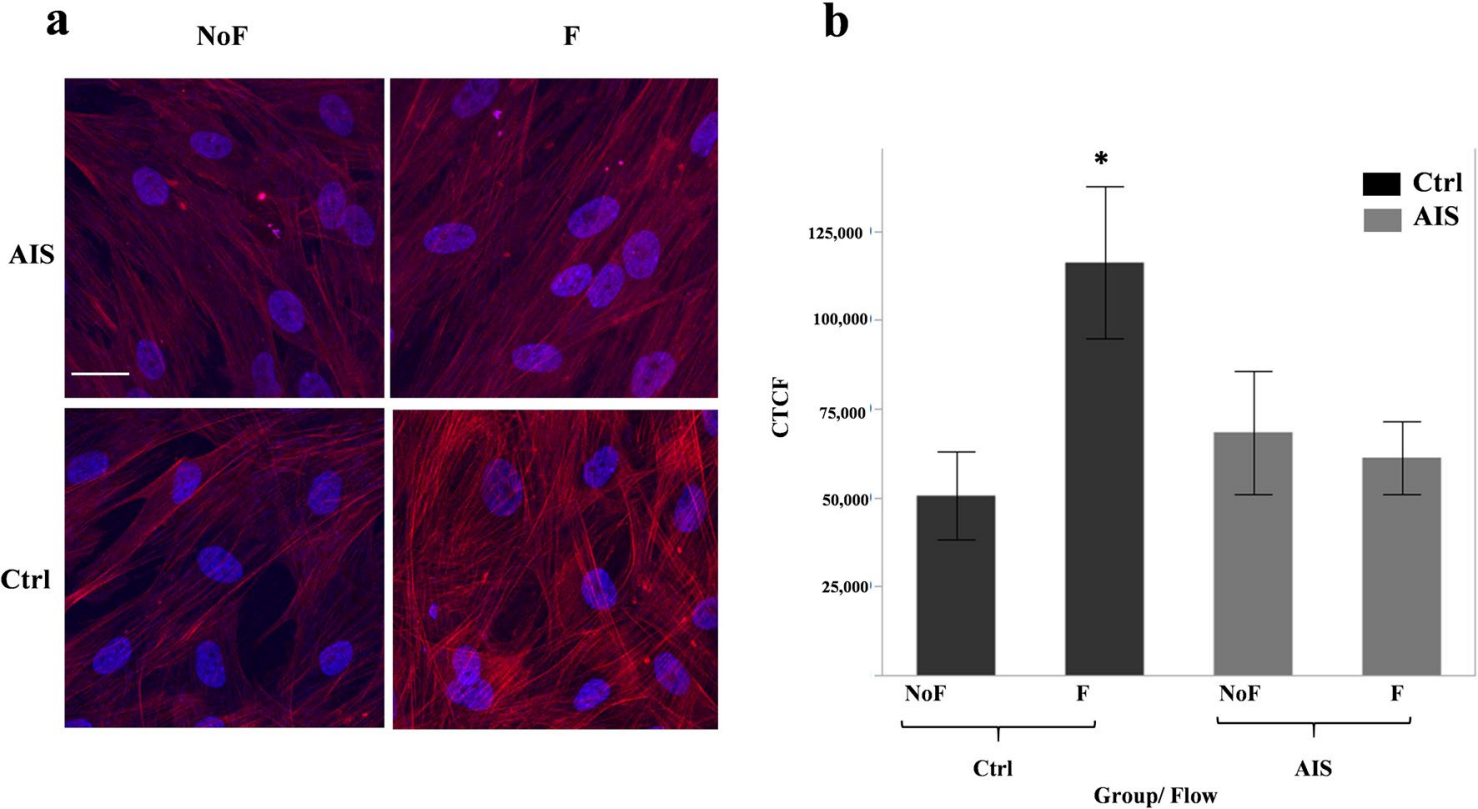
Flow induces actin rearrangement in control osteoblasts but not AIS. (**a**) Immunofluorescence micrographs of AIS and control primary osteoblast cells after 20 h of fluid flow application in comparison to their static counterparts. F-Actin is stained with Phalloidin (red) and nucleus with Hoescht (blue). White scale bar, 25 µm. (**b**) Data represents the mean ± SEM of corrected total cell fluorescence (CTFC) which have been measured using Image-J software at 5 fields of view per sample (20 per group) and normalized to the background of each sample. n = 4 per group, Statistical significance was determined by Student T-test using JMP-14.

Mechanotransduction induced actin remodeling in bone cells as previously reported^19^. Fluid shear stress applied to osteoblasts is expected to induce actin reorganization into thicker, structured contractile stress fibers^20,21^. Disruption of the actin cytoskeleton negatively affects cellular mechanosensitive responses, while increased actin polymerization promotes osteogenic differentiation^21^. To test the response of actin filaments to fluid flow, we measured the intensity of fluorescently stained actin relative to their corresponding background in mechanically stimulated (flow, F) and no mechanical stimulation (no flow, NoF) groups comparing AIS primary osteoblasts to control cells. Application of an oscillatory fluid flow induced F-actin remodeling and reorganization in control cells. This is consistently visible across images acquired from control cells subjected to flow. In contrast, AIS osteoblasts in the same condition, showed no actin response (Fig. 2a). Moreover, following flow application, control osteoblasts showed a significant increase in F-actin intensity compared to no flow (2.3-fold increase, *P* = 0.009), while the F-actin intensity in flow subjected AIS cells remained unchanged (Fig. 2b).

### Fluid flow does not increase the rate of wound healing/cell migration in AIS cells

The orientation of primary cilia normally changes in response to wound healing stimulation, turning perpendicular to the leading edge of a migrating cell after 10 h in culture^22^ as if pointing towards the direction of migration^23^. This dynamic change in cilia is a consequence of tightly regulated assembly and disassembly, which is mediated through F-actin dependent mechanisms^22^. Furthermore, using cells derived from a mouse model, mechanical stimulation was reported to accelerate wound closure by about 50%^24^.

**Figure 3:**
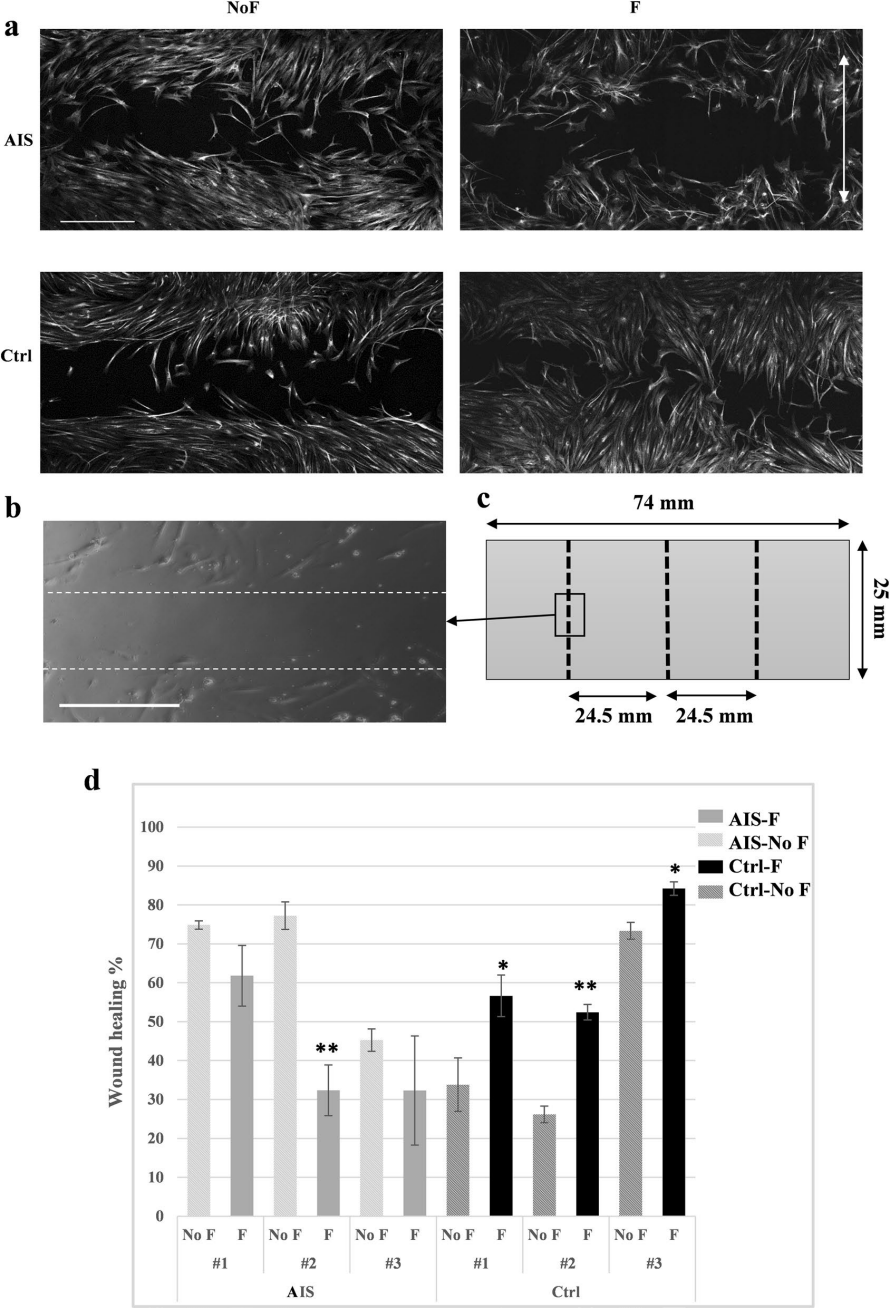
Fluid flow does not increase the rate of wound healing/cell migration in AIS cells. (**a**) Grayscale immunofluorescence micrographs (Objective 10×) of wounded monolayers of AIS and control primary osteoblast at T20h with (F) or without (NoF) flow application. Cells are stained for their nucleus and actin filaments. White scale bar, 500 µm. The double-headed arrow indicates the direction of flow. (**b**) An example of a T0 image that shows live cells immediately after applying the scratch (Objective 20×), the dotted white lines indicate the boundaries of the scratched line, Scale bar, 500 µm. (**c**) The schematic view of the one well chamber slide, dotted black lines show the three scratches and their positioning. We studied three scratched per sample, and three fields of views per scratch. The study was performed independently on cell samples from 4 control and 4 AIS donors (n = 4 per group). (**d**) Data represents the mean ± SEM of wound healing percentages for IS and control cells. Each no flow (NoF) condition sample was compared to their corresponding flow condition (F) using Student T-test using JMP-14. *p value < 0.05, ** *p* value < 0.005.

To assess whether AIS cells show impaired actin dynamics, we examined the rate of wound healing using a scratch test applied to the cultured osteoblast monolayer surface in AIS and control cells, with and without the influence of fluid flow. We scratched each sample in three straight lines using a sterile pipette tip, recorded images of the T_0_ scratched area for each sample and compared that to the same area after 20 h (T_20_) of applying fluid flow (F) or no flow (NoF). The uncovered area of each T_20_ scratch was measured in comparison with its baseline value (T_0_) to calculate the percentage of wound healing. Considering the natural variability of migration rates that is expected from human primary cells, we decided to compare each AIS or control cell only to its own static counterpart rather than comparing the whole AIS group vs. controls (Fig. 3). In osteoblasts obtained from control subjects, the cells in all T_20_, F and NoF samples migrated into the scratched area, with a significant increase (*P* < 0.05) in the rate of wound healing under fluid flow (56.6%, 52.4% and 84.2% wound closure, per scratch) compared to their static counterparts (33.8%, 26.2% and 73.3% wound closure). Although wound healing was also visible at T_20_, in both F and NoF AIS osteoblast samples, fluid flow application not only did not enhance the healing process, but reduced the rate of healing (flow induced wound closures were 61.8%, 32.4% and 32.3% versus 74.8%, 77.2% and 45.3% without flow). AIS osteoblasts migrated through the scratched gap faster in stationary conditions (NoF), with one AIS case showing a significant difference (77.2% NoF vs. 32.4% F, *P* < 0.005) (Fig. 3d). Therefore, flow application on AIS osteoblasts did not promote cell migration as expected, but instead suggests an inhibitory effect.

### AIS cellular orientation in response to directional flow is impaired

Nuclear positioning and orientation relative to the leading edge of a moving cell contributes to the polarized, asymmetrical task of cellular migration. Multiple cytoskeletal elements including cilia and actin filaments have been shown to associate with movement and orientation regulation of the nucleus and the cell as a unit. Nuclear rotation and orientation have been hypothesized to be the consequence of cytoskeleton rearrangement^25^.

**Figure 4:**
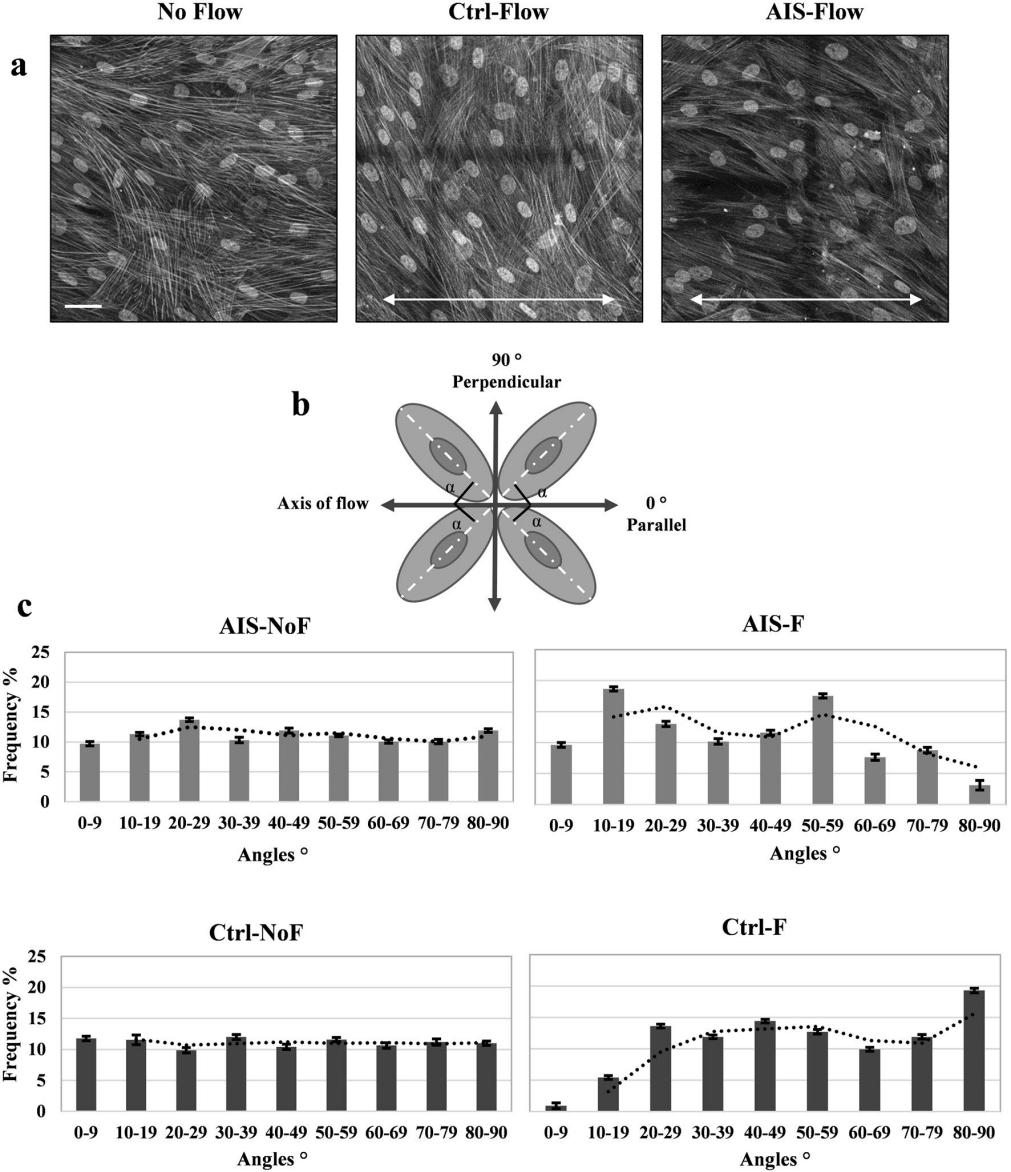
Cellular orientation adjustment relative to direction of flow varies between control and AIS primary osteoblasts. (**a**) Grayscale immunofluorescence micrographs (Objective 63 oil) of stained nucleus and actin F filaments, to show how the cells orient themselves relative to the axis of flow (20 h), in 4 fields of view for each sample. Cell samples from 4 control and 4 AIS patients were studied (n = 4 per group). White scale bar, 25 µm. Two headed white arrows show the direction of oscillatory fluid flow application. (**b**) For each sample 500–750 angles between the longest axis of the nucleus and axis of flow (α) were measured in 5 × 4 tile images (20 fields of view) as shown in this schematic figure (adapted from Yao and Wong (2015) Cells. J. Biomech. Eng. 137, 020,907). (**c**) Data represents the frequency of cellular alignment distribution in percentage after 20 h of 1 Hz flow application. Trend line of moving average is shown as dotted lines.

It has been reported that directional mechanical stimulation could affect the orientation and morphology of mesenchymal stem cells (MSCs) in culture^26^. After observing the defects in cilia length adjustment and actin filament rearrangement in response to flow, we decided to look at the orientation and positioning of cultured primary osteoblasts following 20 h of oscillatory fluid application (AIS patients compared with osteoblasts from controls, Fig. 4a). We measured the angle between the long axis of the nucleus relative to the direction of applied flow (Fig. 4b) in 500–750 cells across 20 different fields of view per sample (n = 4 per group). In theory, random cell orientation would result in one-third of the cells being oriented with their long axis ± 30° from any random line drawn through the field of cells^20^. This means that in static conditions, cellular population orientation relative to an arbitrarily selected line will be divided in 3 groups: ~ 33% between 0–30°, ~ 33% between 30–60° and ~ 33% between 60–90°. As presented in Table 1 and Fig. 4c, no flow samples in both AIS and control groups followed the expected random distribution. After flow application (20 h, 2 Hz), 41.1% of control cells were positioned in angles between 60° to 90° relative to the axis of flow. This shift of distribution is visible in the bar charts of Fig. 4c. While flow application seemed to alter the distributions of orientation among AIS cells, only 19.5% were shifted towards perpendicular angles relative to the axis of strain at the end of 20 h flow. Our results show that AIS osteoblasts fail to align themselves normally in response to the axis of a bidirectional oscillatory flow, which is a prominent type of load induced force affecting bone cells in vivo^21^.

**Table 1:** The distribution of α angle in control and AIS osteoblast with or without flow application.

	AIS—No Flow	AIS—Flow	Ctrl—No Flow	Ctrl—Flow
0–30°	34.7%	41.24%	33.17%	19.95%
30–60°	33.29%	39.27%	33.99%	38.97%
60–90°	32.01%	19.49%	32.84%	41.08%

### Fluid flow does not induce secretion of VEGF or PGE2 in the medium of cultured AIS osteoblasts

**Figure 5:**
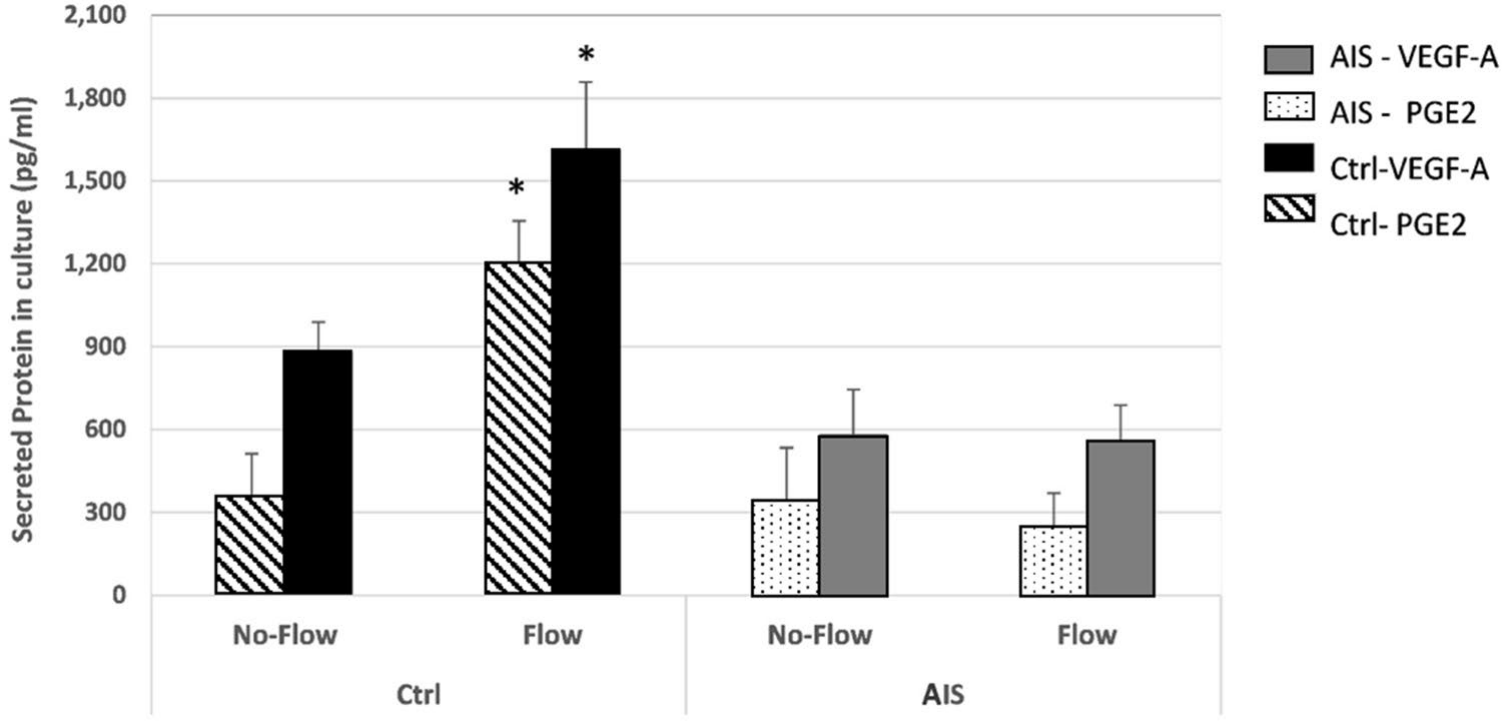
Fluid flow does not induce neither VEGF-A nor PGE2 secretion in AIS osteoblast cells. The quantity of secreted VEGF-A and PGE2 were measured in the media of both AIS and Control cultured cells, following 20 h fluid flow application in comparison to their stationary counterparts. N = 4 per group, each standard error bar is constructed using 1 standard error from the mean in JMP-14 software. * Student T-test *p* value < 0.05. Legend; Solid bars: VEGF-A, Patterned bars: PGE2, Darker bars: Controls, Lighter bars: AIS.

Prior work using mouse osteoblasts have shown that pathways involved in regulation of the actin cytoskeleton and VEGF signalling are activated under mechanical induction^27^. PGE2 is an important bone remodeling factor that has been shown to dramatically increase in response to fluid flow in osteoblasts^28^. Also, PGE2 and VEGF have been shown to positively affect each other’s expression in human endothelial cells^29^. This prompted us to investigate both VEGF and PGE2 secretion in the media of cultured AIS and control osteoblasts. We found that in control osteoblasts, fluid flow application during 20 h significantly increased secreted VEGF by an average of 83.1% (Student t-test, *p* = 0.018), and PGE2 by an average of 233.6% (Student t-test, *p* = 0.007) (Fig. 5). However, there was no significant change in secretion of VEGF nor PGE2 by AIS osteoblasts, further suggesting an impaired mechanotransduction pathway.

## Discussion

This exploratory study further characterizes mechanosensory abnormalities occurring under mechanical strain in AIS osteoblasts, building on our previous findings showing an elongated primary cilia phenotype in AIS stationary cells, along with an altered molecular mechanosensory response^10^. We found that control osteoblasts modify the length of their primary cilium in response to fluid flow application in a time dependent manner. However, AIS osteoblasts exhibited a different response than control cells. In control cells, short-term flow (1.5 h) reduced the average length of cilia, while continuous application of the same flow regimen induced an increase in ciliary length. In contrast, AIS osteoblasts increased the length of their cilia following short-term fluid flow, while the continuous flow application had no significant effect on their average ciliary length. These results show that the cilia abnormalities described initially by the works of Oliazadeh et al*.*^10^ represent a more dynamic process varying in function of mechanical circumstances.

The relationship between mechanical strain and bone morphology is complex, with adaptive changes on molecular, cellular and tissue levels. It is known that different types of mechanical stimulation can activate different molecular pathways; stretch, compression, gravity, vibration and fluid shear stress are all physiological forces but they differ in their effects and mechanisms (reviewed in^30^). Even the same type of stress can be applied with different magnitudes, durations and frequencies which can induce different cellular responses. For example human fetal osteoblasts are responsive to pulsatile shear stresses but not to steady or oscillatory ones^31^. As a mechanosensory organelle, increase or decrease in the length of cilia, in response to mechanical stimulations, is a cellular attempt to adjust its mechanical sensitivity. Cultured osteoblasts stimulated with a long period of oscillatory fluid flow (up to 5 days) have been shown to shorten their ciliary length^18^. Cilia length adjustment in response to different flow application regimens and its importance on downstream cellular adaptions has been shown in several human cell types including osteoblasts^14,18^. Longer cilia are shown to be more sensitive to mechanical stimulation^32,33^.

The failure of AIS osteoblasts to adjust their ciliary length in function of the applied mechanical stimulation (i.e. short- or long-term fluid flow) suggests possible ciliary defects underlying AIS pathogenesis. Cilia length regulation and maintenance is the result of a precise balance between processes involved in its assembly and disassembly. Stimulation of cyclic AMP and subsequent increased activity of PKA has been shown to lengthen cilia in mammalian epithelial and mesenchymal cells, through increased trafficking of anterograde intraflagellar transport (IFT) complex^14^. Indeed, IFT is a tightly conserved evolutionary system, which is responsible for transferring molecules to and from the tip of the cilium as an organelle extended out of the cytoplasm. The accumulation or increased activity of the anterograde IFT system leads to elongation of cilia whereas their reduced mobility results in shorter cilia^34^. Fluid shear-mediated deflection of the longer primary cilia then stimulates cAMP reduction inside the cell, creating a regulatory feedback loop which shortens the cilia again^35^. Therefore, Gi-coupled receptor signalling dysfunction and the following disturbance of intracellular cAMP previously reported in AIS patients^36–38^ might explain ciliary length abnormalities observed in AIS osteoblasts. Incapable of adjusting the length of their cilia accordingly, AIS osteoblasts are not able to transfer proper information to the cell regarding the type and scale of the introduced mechanical stimulation, compromising the adaptive nature of bone to surrounding forces and perhaps leading to structural abnormalities in the bone. There could be a causative link between cilia length misregulation and AIS, as is the case in some types of cancers^22^.

We also investigated characteristics of the cytoskeleton in AIS osteoblasts exposed to fluid flow. Application of an oscillatory fluid flow during 20 h consistently induced actin rearrangement in control osteoblasts, changing them to thicker and more intertwined fibrillar structures with significantly higher intensities. These alterations following mechanical loading cause rearrangement of actin filaments that enhance mechanical resistance of the whole cell^39^. This drastic change of actin filaments is completely missing in AIS osteoblasts under similar flow conditions.

Our functional evaluation of AIS osteoblasts showed that fluid flow application does not accelerate their migration rate, compared to static culture condition (NoF). Surprisingly, the AIS osteoblasts healing process was faster under stationary conditions, as demonstrated in the scratch test, while control osteoblasts responded positively to fluid flow stimulation, closing the gap more efficiently under flow. Cilia not only sense the mechanical strain but also have been shown to play a crucial role in the cellular migration process. The primary cilium points to the direction of migration and guides the cell through the extracellular matrix by complex and closely regulated molecular signaling pathways that are not yet truly understood. Ciliary interactions with ECM, Wnt pathway, and polarity signalling have all been suggested to play a role in this process^23^.

Cellular responses to directional flow are also determined by cell shape, strongly suggesting that cytoskeleton and adhesive structures play as an internal compass against which flow is measured^40^. Control osteoblasts were observed to begin shifting their orientation perpendicular to the direction of strain. After 20 h of flow application, the α angle of 41% of Ctrl-F group was measured between 60° to 90° compared to only 33% in control NoF group. While mechanical stimulation also disturbed the normal angle distribution in AIS-F cells, no particular pattern was observed in their orientation in response to flow (Table 1). Previous studies have shown that the angle between flow direction and the cell axis, which is defined by cell shape and F-actin, dictates these flow responses, suggesting a central role for cell alignment in the response to shear stress^40^.

Following the observed abnormalities in actin remodeling responses, flow induced migration and positioning of AIS osteoblasts under flow, we decided to test the possibility of a disturbed VEGF signaling. As expected, the quantity of secreted VEGF in the media of control-F group almost doubled after 20 h of flow induction, while fluid flow application on AIS cells did not affect the amount of secreted VEGF in their culture media. The expression of VEGF in osteoblasts has also been shown to increase by PGE2^41^. Prostaglandin E2 is one of the potent mediators of mechanical induced bone remodeling which dramatically increases in bone cells following fluid flow stimulation^28,42–47^.

In the present exploratory study, we acknowledge some limitations. The relatively small sample size of AIS cases tested, and the selection of only severe scoliosis cases should be mentioned. Secondly, it remains to be investigated if other musculoskeletal cell types harboring cilia will exhibit the same dysfunction as evidenced in AIS osteoblasts. Finally, the molecular mechanism regulating VEGF and PGE2 signaling and/or their secretion in AIS remains to be characterized and represents an unexplored frontier in the field of scoliosis.

As a condition without a well-defined cause, AIS treatments are focused on correction of the symptomatic spinal curvature while the underlying pathomechanism remains unknown. In order to develop innovative treatments addressing the root cause(s) of AIS, the molecular events underlying its pathophysiology must be understood at the biological level. To this end, we studied the cellular characteristics of AIS patient bone. Collectively, our data further support the presence of a disturbed mechanotransduction in AIS osteoblasts that goes beyond morphological changes in cilia. A compromised actin dynamics in the context of AIS, can also affect ciliogenesis, resulting in a wide variation in ciliary lengths and a systemic mechanotransduction impairment^22^. From a clinical point of view, our results could explain the differential bracing outcomes among AIS patients as recently demonstrated by the works of Beauséjour et al.,^48^.

## Materials and methods

### Patient enrolment for sample collection

Samples used for this study were derived from specimens obtained intraoperatively from AIS patients and trauma control subjects. This study was approved by the institutional review boards of Sainte-Justine University Hospital (project #2380), Montreal Children’s Hospital, Shriners Hospital for Children in Montreal. A signed informed consent was obtained from the parents or legal guardians of each minor subject and assent was obtained from each participant. All experiments were performed in accordance with respective guidelines and regulations. Clinical and demographic details of recruited subjects for bone tissue harvest are listed in Table 2.

**Table 2:** Clinical and demographic data of patients.

AIS Patients					
ID	Sex	Age (years)	Diagnosis	Cobb angle (°)	Curve type
593	F	14.1	AIS	80–64	rTlL
1642	F	17.8	AIS	37–68-39	lTrTlL
1653	F	11.2	AIS	68	rT
1654	F	16.2	AIS	67–71	rTlL
1659	F	16.0	AIS	50–89	rTlTL

Control Patients					
ID	Sex	Age (years)	Trauma Diagnosis	Anatomic site of specimen collection	Side of specimen collection
T13	F	18.7	Hip dislocation	Tibia	Left
T14	F	11.6	Osteochondromatosis	Proximal tibia	Right
T19	F	15.5	Clubfoot	Tibia and fibula	Right
T22	F	14.0	Patella dislocation	Tibia	Left
T45	F	15.2	Inegality of lower limbs	Femur and tibia	Left

rTlL: Right Thoracic-Left Lumbar; lTrTlL: Left Thoracic-Right Thoracic- Left Lumbar ; rT: Right Thoracic ; rTlTL: Right Thoracic- Left Thoracolumbar.

### Cell culture

Primary osteoblast cultures (passages 2–3) were generated from bone biopsies obtained intraoperatively from AIS and trauma patients as previously reported^10^. Briefly, bone specimens were extracted from vertebrae (varied from T3 to L4) of AIS patients or other parts of skeleton (tibia or femur) of non-scoliotic trauma cases. After cutting the bone to smaller pieces, they were incubated in cell culture media [αMEM, 10% fetal bovine serum (FBS), 1% penicillin/streptomycin (Invitrogen Life Technologies, ON, Ca)] at 37 °C in 5% CO_2_ for a month. Emerging primary osteoblasts were then separated by trypsinization and characterized using a mineralization assay and RT-qPCR expression analysis for osteoblast markers (Supplemental Information and Supplementary Fig. S1). To promote ciliogenesis, cells were washed in sterile PBS upon confluence and incubated in differentiation media (with reduced FBS to 1%) for 48 h. The 48 h period was chosen based on previous studies, to possibly minimize the differences between cilia length of controls and AIS. Cells were washed with PBS after cilia induction and incubated in regular media right before starting all our experiments.

### In vitro fluid flow stimulation

For shear stress experiments, each sample was divided between two 1-well chamber slides (Thermo Fisher scientific, Nunc Lab-Tek, MA, USA) at a density of 3 × 10^5^ cells per well in complete medium (αMEM + 10% FBS + 1% penicillin/streptomycin). Upon reaching 80% confluency, the medium was removed, the cells were washed with warm, sterile PBS and then transferred to a starvation medium. After 48 h cells were washed again and transferred back to 2 ml regular medium immediately before they were subjected to oscillatory fluid flow using a double-tier rocking platform, as explained in our previous work^10^. In summary, a two-tier rocker with ± 20 degree of maximum tilt angle was housed in a cell culture incubator at 37 °C and 5% CO_2_ for the duration of the flow experiments. Flow samples were sat on the rocker moving at a frequency equal to 2 Hz, while no flow control cells were kept in the upper shelf of the same incubator during the experiment. Fluid shear stress patterns were applied to cells in a predictable, controlled, and physiologically relevant manner in a magnitude of 1 Pa at the middle of the dish as demonstrated previously^10^. We applied a 1 [Pa] shear stress (the magnitude at the center of the dish when the dish was horizontal) in 1 [Hz] frequency, which corresponds to a Womersley number of 6.5. The biomechanical parameters were chosen to be physiologically relevant based on the reported frequency spectra of forces affecting the human hip during walking, (1–3 Hz)^49^, the Womersley number estimated for cerebrospinal fluid motion in the spinal cavity (5–18)^50^. The Womersley number takes into account the effect of viscosity and shear stress exerted on the cell and is widely used in biomechanical studies involving pulsating fluid flow^51^. The value of Womersley number ranges from 5 to 18 in fluid motion of cerebrospinal fluid in the spinal cavity^52^. We designed our experiment such that the Womersley number experienced by the cells is equal to 6.5, which is well within the expected range in vivo (see Zhou et al. 2010^53^ for details of calculation).

### Immunofluorescence staining

Cells were washed with PBS, fixed with 4% paraformaldehyde (PFA) in PBS buffer for 10 min at room temperature, washed again with 1% bovine serum albumin (BSA) in PBS, and then permeabilized with 0.1% Triton-X-100 in PBS for 10 min at room temperature. After two washes, the cells were blocked in 5% BSA in PBS for 1 h at room temperature. For cilia staining, cells were incubated with anti-acetylated α -tubulin antibody (Invitrogen Life Technologies, ON, CA) diluted (1:1000) in 3% BSA-PBS, overnight at 4 °C. The following day, after three washes, the cells were incubated for 1 h at room temperature with Alexa Fluor 488 conjugated goat anti-mouse secondary antibody (Invitrogen). After three washes, 1 μg/ml dilution of Hoechst (Sigma-Aldrich, ON, CA) in 1% BSA-PBS was used to stain the nucleus at room temperature for 10 min. F-actin was stained with Alexa Fluor 555 Phalloidin (Invitrogen), dilution (1:40) in 1% BSA-PBS incubation at room temperature for 20 min.

### Scratch/wound healing test

Osteoblasts from both AIS and control groups (n = 4 per group) were cultured in one chamber slide dish (Thermo Fisher Scientific, Nunc Lab-Tek, MA, USA). Upon reaching 80% confluency, and after 48 h of ciliogenesis, three linear wounds were created in each slide, perpendicular to the long edge and equally distanced from each other by scratching the monolayer using a sterile 200 μl pipette tip^54^. Cells were then washed with PBS to remove floating cells and debris before being transferred to regular warmed media. The scratched areas in live cells were imaged using ENVOS FL Microscopy (ThermoFisher Scientific, MA, USA), objective 20x. These images were then used to calculate the T_0_ area of the wounds in corresponding samples. After flow applications, immunofluorescence staining and imaging, the degree of wound closure was measured manually as the percentage of the area covered by migrating cells at T_20_ compared to the initial wound at T_0_, using Fiji software^55^. We evaluated three wounds per sample, and three fields of view per wound. For each scratch, we averaged the percentage of cells that migrated into the scratch area at T_20_ relative to T_0._ For each group (AIS or control), we compared the flow versus no flow scratches that were in the same relative positions on the slide (Fig. 3c,d).

It should be noted that the shear stress is not uniform across the culture chamber and varies quadratically with position (see Eq. 9 of Zhou et al.^53^). For the case where the shear stress at the center of the dish is 1 [Pa] when horizontal, the shear stress at the location of two other scratches are estimated to be around 0.3 [Pa].

### Confocal microscopy and image analysis

Images were captured on a Leica Confocal TCS-SP8 using 63x (oil) or 10 × objectives with 1024 × 1024 pixels resolution. Each sample was examined in stitched 5 × 4 or 5 × 5 tile images, (covering 20 or 25 fields of view). Maximum projections of the Z-stacks were used for primary cilium length and actin intensity measurements, which was done using the Image J software (NIH). To be able to measure the intensity of fluorescent stained actin as an indicator of protein quantity, all related images were acquired using the same microscope under identical settings (i.e. laser intensity, acquisition time, resolution, etc.). Corrected total cell fluorescence (CTFC) was calculated by measuring actin intensity normalized to the background of each sample, using Fiji software. For cell alignment analysis, we used the longest axis of the nucleus as the major axis of the cell. The orientation of each cell was determined by measuring the angle (α) between the cell and the axis of flow application manually in the Fiji software. We evaluated cellular orientation in 5 × 4 tile images, i.e. 20 fields of view per sample. The goal of this experiment was to quantify the effect of shear stress on nuclear orientation based on the angle between longest axis of nucleus (as the axis of the cell) and the axis of flow. Cells with round nuclei were omitted from evaluation since all their axes are the same size and they could not serve a purpose in our data based on the parameter of the experiment.

### Vascular endothelial growth factor (VEGF-A) and Prostaglandin E2 (PGE2) measurements

Cell culture media was collected from samples at the end of each fluid flow experiment, aliquoted, labeled and transferred to − 80 °C for later processing. On the day of experimentation, the samples were thawed on ice and centrifuged at 4 °C, 10,000×*g* for 10 min to remove possible cellular debris. VEGF-A levels were measured using the Human VEGF-A Platinum ELISA kit (ThermoFisher Scientific, Waltham, MA, USA), following the manufacturer’s protocol. Absorbance was read at 450 nm, using the DTX880 Multimode Detector (Beckman Coulter, Brea, CA, USA). PGE2 levels were also measured in the same media samples, using the Human PGE2 ELISA kit (Invitrogen Life Technologies, ON, Ca), following the manufacturer’s instructions. Absorbance was read at 405 nm, using the DTX880 Multimode Detector.

### Statistical analysis

All experiments were conducted in replicates, with sample sizes of 4 (N = 4) per study group. All data was analysed using the JMP-14 Statistics Software from SAS Institute (Cary, NC, USA). Student T-Tests were used to determine differences between study groups, and differences were considered statistically significant when *p* values < 0.05.

## Supplementary Information

Supplementary Information.

## Abbreviations

AIS: Adolescent idiopathic scoliosis
VEGF: Vascular endothelial growth factor
OB: Osteoblasts
F-actin: Filamentous actin
NoF group: No flow group
F group: Flow group
MSCs: Mesenchymal stem cells
IFT: Intraflagellar transport
cAMP: Cyclic adenosine monophosphate
PGE2: Prostaglandin E2
