# Supplementary material for: Altered mechanotransduction in adolescent idiopathic scoliosis osteoblasts: an exploratory in vitro study
Source: Sci Rep. 2022 Feb 3;12:1846. doi: 10.1038/s41598-022-05918-0 (PMC8813918; doi:10.1038/s41598-022-05918-0)
Supplement: Supplementary file 1 — Supplementary Information. [file 41598_2022_5918_MOESM1_ESM.pdf]

Altered Mechanotransduction in Adolescent Idiopathic Scoliosis Osteoblasts: An Exploratory In Vitro Study

Niaz Oliazadeh[1,2], Kristen F. Gorman[3], Mohamed Elbakry[1,4] and Alain Moreau[1,2,5*]

[1]Viscogliosi Laboratory in Molecular Genetics of Musculoskeletal Diseases, Saint-Justine University Hospital Research Center, Montreal, QC, Canada.

[2]Department of Biochemistry and Molecular Medicine, Faculty of Medicine, Université de Montréal, Montreal, QC, Canada.

[3]Department of Biological Sciences, California State University, Chico, CA 95929, USA.

[4]Biochemistry Division, Chemistry Department, Faculty of Science, Tanta University, Tanta, Egypt.

[5]Department of Stomatology, Faculty of Dentistry, Université de Montréal, Montreal, QC, Canada.

*Corresponding author: Alain Moreau, PhD, Saint-Justine University Hospital Research Center, Montreal, room 2.17.027, 3175 Cote-Ste-Catherine Road, QC, H3T 1C5 Canada.

Phone: +1 (514)-345-4931 ext: 5722 | Fax: +1 (514)-345-4801

E-mail: alain.moreau.hsj@ssss.gouv.qc.ca

Supplemental Information

Phenotypic characterization of human osteoblasts derived from AIS patients. Notwithstanding the differences reported in cilia length between AIS osteoblasts and control cells, the phenotype of AIS and control osteoblasts was determined by evaluating the cellular proliferation, bone nodule mineralization (Alizarin red assay), and the mRNA expression analysis of key osteogenic markers (Supplementary Figure S1). We did not observe significant differences in proliferation and mineralization rates between groups (Supplementary Figure S1, panels A and B). However, our expression analyzes of key osteogenic markers revealed some differences (Supplementary Figure S1, panels C and D). While no significant changes were observed with RUNX2 and SP7 expression, which

are early-stage osteoblastic markers, we noticed a stronger expression of ALP and SPP1 genes in AIS osteoblasts in both growth and differentiation (mineralization) media. Interestingly, a recent publication reported a significantly greater alkaline phosphatase (ALP) activity in AIS osteoblasts obtained from the convex side of the spinal curve compared with those derived from the concave or non-curve osteoblasts [1]. Unfortunately, the exact origin of the bone samples obtained intraoperatively from our AIS patients was not available in the context of this exploratory study. Of note, higher serum levels of ALP were observed in pre- and post-menarcheal scoliotic girls compared with controls [2]. Regarding the stronger expression in AIS osteoblasts of SPP1 gene, which encodes for osteopontin (OPN), several reports have shown by different indirect approaches that elevation of OPN could be associated with scoliosis onset and spinal deformity progression [3-5]. Finally, the decreased expression of OCN gene observed in our AIS osteoblasts (surgical AIS cases) is supported by the findings of Chen *et al*., reporting that the serum osteocalcin (OCN) levels were significantly decreased in AIS patients exhibiting a severe scoliosis [6]. It remains unclear at this stage and beyond the scope of this exploratory study, whether the expression changes observed in some of osteogenic markers in AIS osteoblasts result from alterations of cellular mechanotransduction in AIS and additional experiments must be undertaken to address this possibility.

Materials and Methods

Preparation of primary human osteoblasts. Primary osteoblast cultures (passages 2-3) were generated from bone biopsies obtained intraoperatively from AIS and non-AIS patients. Briefly, bone specimens were extracted from vertebrae (varied from T3 to L4) of AIS patients or other parts of the skeleton (tibia or femur) of non-scoliotic cases. The bones were cut into small pieces of 1-3 mm^3 using a sterile stainless steel surgical blade. The pieces of bone were washed in serum-free media three times and then serially digested twice by incubating in 1mg/ml type-I collagenase for 20 min followed by 2 hours of further incubation (Sigma-Aldrich, St-Louis, MI, USA) at 37°C. The digested bones were washed three times in serum-free medium, with vigorous hand shaking and then cultured in alpha-MEM with 20% FBS and 1% antibiotic-antimycotic solution.

Osteoblast differentiation of primary osteoblasts was induced by culturing the cells in alpha-MEM media supplemented with 10% FBS, 100nM dexamethasone, 20mM glycerophosphate, 50μM ascorbate-2-phosphate and 50nM vitamin D3.

Alizarin red staining of osteoblast cultures. The mineralized osteoblast cultures (at day 14) were fixed in 70% ethanol for 1 hour at 4°C. The cells were then stained with 0.2% Alizarin red S in 2% ethanol for 15 minutes. The cells were washed five times with water and dried at 37°C, then photographed.

RNA extraction for expression analyzes. RNA was extracted from the osteoblasts using the TRIzol method (Thermo Fisher Scientific, Waltham, MA, USA).

Expression analysis by quantitative real-time PCR. cDNA was synthesized from the extracted mRNA samples using a PCR thermocycler (T3000 Thermocycler, Biometra, Montreal Biotech Inc, Montreal, QC, Canada) and All-In-One 5X RT MasterMix (Applied Biological Materials Inc., Richmond, BC, Canada) by following manufacturer's instructions. The resulting cDNA samples were stored at -20°C. Amplification of the qPCR reactions was performed using the QuantStudio™ 3 instrument (Thermo Fisher Scientific) and TaqMan Advanced mRNA Assays (Thermo Fisher Scientific). The probes for each mRNA used to detect the mRNA expression were: alkaline phosphatase (ALP)/ Assay ID: Hs01029144_m1; Runx2/ Assay ID: Hs00231692_m1; osteopontin (SPP1)/ Assay ID: Hs00959010_m1; osteorix (SP7)/ Assay ID: Hs00959010_m1; osteocalcin (OCN)/ Assay ID: Hs01587814_g1; and GAPDH/ Assay ID: Hs02786624_g1 (housekeeping gene control). All qPCR experiments were performed in duplicate for each time point and the mean of the obtained cycle thresholds (CT) was used for calculations.

Supplementary Figure Legend

Supplementary Figure S1. Mineralization and gene expression analysis of osteoblast markers. Mineralization assay of AIS osteoblasts (panel A) or control osteoblasts (panel B), cultured in growth medium (α –MEM) or mineralization medium are illustrated at day 1 and day 14 (stained by alizarin red S to reveal mineralization nodules). Expression

of osteogenic markers was performed with total RNA extracted from AIS osteoblast (panel C) and control osteoblast (panel D) at different time points in growth and differentiation media. Expression of Alkaline phosphatase (ALP), RUNX2, Osteopontin (SPP1), Osterix (SP7) and Osteocalcin (OCN) were examined by qPCR, using GAPDH as the housekeeping gene. All expression analyzes were performed in duplicate for each time points.

Supplementary references

1. Pearson, M.J., et al. Evidence of Intrinsic Impairment of Osteoblast Phenotype at the Curve Apex in Girls With Adolescent Idiopathic Scoliosis. *Spine Deform.* **7**(4): 533-542 (2019).

2. Kulis, A., et al. Participation of sex hormones in multifactorial pathogenesis of adolescent idiopathic scoliosis. *Int Orthop*. **39**(6): 1227-36 (2015).

3. Xie, N., et al. Does elevated osteopontin level play an important role in the development of scoliosis in bipedal mice? *Spine J*. **15**(7): 1660-4 (2015).

4. Yadav, M.C., et al. Ablation of osteopontin improves the skeletal phenotype of phospho1(-/-) mice. *J Bone Miner Res*. **29**(11): 2369-81 (2014).

5. Akoume, M-Y., Franco, A., Moreau, A. Cell-based assay protocol for the prognostic prediction of idiopathic scoliosis using cellular dielectric spectroscopy. *J Vis Exp*. (80): e50768 (2013).

6. Chen, H., et al. Abnormal lacuno-canalicular network and negative correlation between serum osteocalcin and Cobb angle indicate abnormal osteocyte function in adolescent idiopathic scoliosis. *FASEB J*. **33**(12): 13882-13892 (2019).

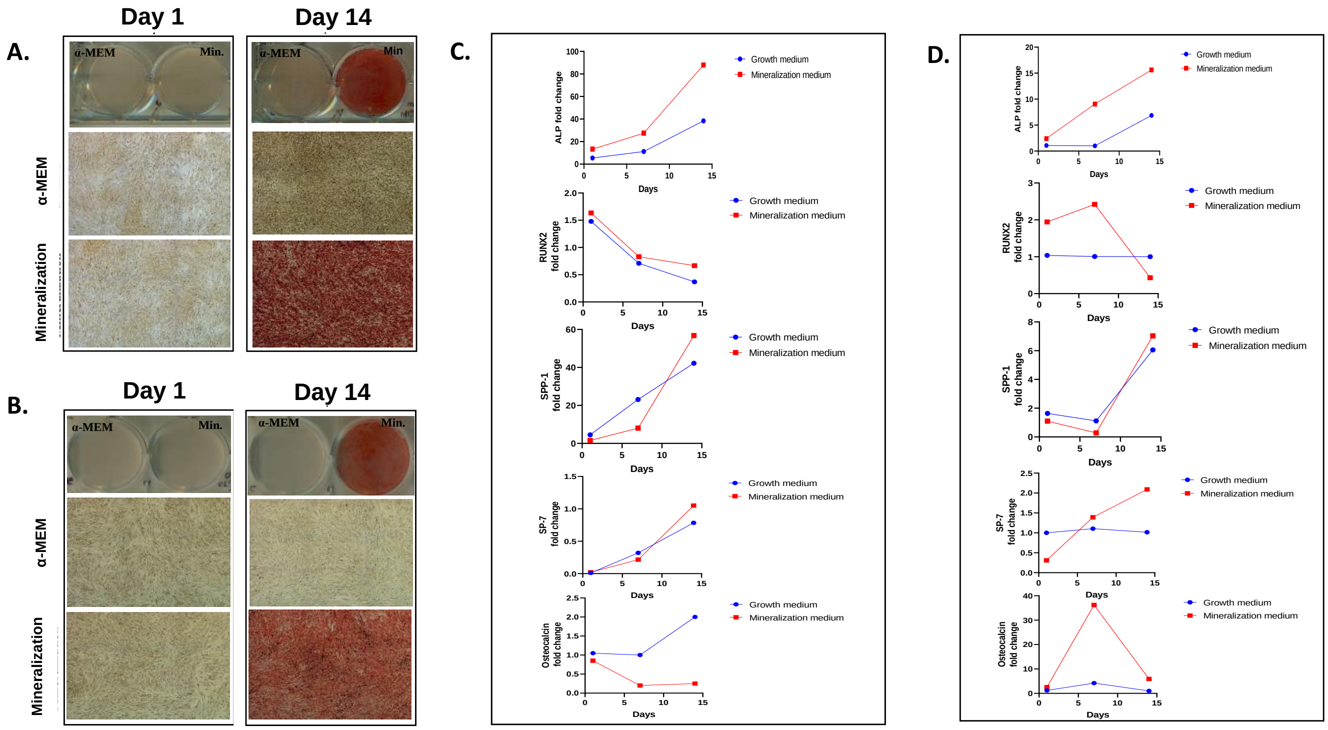

Supplementary Figure S1.
